# The Novel Oral Drug Subetta Exerts an Antidiabetic Effect in the Diabetic Goto-Kakizaki Rat: Comparison with Rosiglitazone

**DOI:** 10.1155/2013/763125

**Published:** 2013-05-08

**Authors:** Danielle Bailbé, Erwann Philippe, Evgeniy Gorbunov, Sergey Tarasov, Oleg Epstein, Bernard Portha

**Affiliations:** ^1^Laboratoire B2PE (Biologie et Pathologie du Pancréas Endocrine), Unité BFA (Biologie Fonctionnelle et Adaptive), Equipe 1, Université Paris-Diderot et CNRS EAC 4413, Bâtiment Buffon, 5éme étage, Piéce 552A, 4, rue Lagroua Weill Hallé, Case 7126, 75205 Paris Cedex 13, France; ^2^OOO “NPF “MATERIA MEDICA HOLDING”, 3rd Samotyochny Per., 9, Moscow 127473, Russia

## Abstract

The aim of the present study was to evaluate the potential antidiabetic effects of two-component drug Subetta and its components (release-active dilutions of antibodies to **β**-subunit insulin receptor (RAD of Abs to **β**-InsR) and to endothelial nitric oxide synthase (RAD of Abs to eNOS)) in Goto-Kakizaki (Paris colony) (GK/Par) diabetic rats. Subetta was administered orally for 28 days once daily (5 mL/kg) and compared to its two components (2.5 mL/kg), Rosiglitazone (5 mg/kg), and vehicle (5 mL water/kg). At day 28, fasting plasma glucose levels were significantly decreased only in Subetta and Rosiglitazone groups as compared to vehicle (*P* < 0.01): 147 ± 4 mg/dL and 145 ± 4 mg/dL and 165 ± 4 mg/dL, respectively. The data of glucose tolerance test showed that Subetta and RAD of Abs to **β**-InsR (similar to Rosiglitazone) prevented significantly (*P* < 0.01) the age-related spontaneous deterioration of glucose tolerance as seen in the control group. Subetta and RAD of Abs to **β**-InsR did not significantly modify the glucose-induced insulin secretion. Chronic administration of Subetta and RAD of Abs to **β**-InsR improves glucose control, to an extent similar to that of Rosiglitazone. We hypothesize that Subetta and RAD of Abs to **β**-InsR mostly act via an insulin-sensitizing effect upon target tissues.

## 1. Introduction

According to the WHO (2012), more than 347 million people worldwide suffer from diabetes mellitus, and among these 90% have type 2 diabetes. That is why type 2 diabetes is ranked high in prophylactic, therapeutic, and rehabilitation programs worldwide. Despite the high efficacy of per oral antidiabetic drugs in treatment of type 2 diabetes, there are a number of limitations associated with their side effects (hypoglycemia, heart failure, body weight gain, lactic acidosis, low tolerability of some drugs, necessity of multiple-dose administration, etc.). The search for new targets, as well as development of innovative approaches for effective and safe action on these targets remains a topical issue. The use of the release-activity phenomenon, which consists in the modifying action exerted by specifically processed ultradilutions on the starting substance [1, 2], could lay the foundation for one of those innovative methods. The drugs of this class containing the so-called release-active dilutions of antibodies [3] demonstrated a fundamentally new pro-antigen (cotargeted with antigen) targeted activity, based on the ability of release-active dilutions of antibodies to modify the nature of antigen-target (molecule-target) interaction via the mechanism of conformational modification. The efficacy and safety of the drugs were intensively studied and proved in different experimental models and in clinical studies as well [1, 4–18].

Subetta contains release-active dilutions of antibodies to *β*-subunit insulin receptor and antibodies to endothelial NO synthase. In experimental model of streptozotocin-induced diabetes Subetta showed pronounced antihyperglycemic activity, which is comparable to that of the reference drug Rosiglitazone: Subetta decreases high plasma levels of glucose, urine levels of ketone bodies and improves glucose uptake in peripheral tissues [7]. Toxicological studies proved a high safety of the drug. The aim of the present preclinical study was to evaluate the potential antidiabetic effects of Subetta and its components in GK/Par diabetic nonobese rats.

## 2. Materials and Methods

Male diabetic (GK) Goto-Kakizaki rats were obtained from the Paris colony (GK/Par), maintained at the University Paris-Diderot animal core, in accordance with accepted standards of animal care as established in the French National Center for Scientific Research guidelines. The characteristics of the adult GK/Par rats have been described previously [19]. Adult male animals (10-week old) were used for this study.

The animals were kept in animal room under artificial light from 8 am to 8 pm. All animals were fed *ad libitum* with a commercial pelleted chow (diet 113, SAVE, Villemoisson-sur-Orge, France) and had free access to tap water.

Animals were assigned randomly to six different groups (*n* = 12 in each group). There was no statistically significant difference among groups with respect to body weight and blood glucose on Day 0 (d0).

The first group (PGK1 group) was given release-active dilutions (ultrahigh dilutions) of antibodies to *β*-subunit insulin receptor (RAD of Abs to *β*-InsR) (OOO “NPF “MATERIA MEDICA HOLDING”, Moscow, Russia) (2.5 mL/kg body weight) once a day, as a solution in distilled water (2.5 mL/kg body weight), so that the total volume administered 5 mL/kg body weight. The second group (PGK2 group) was given release-active dilutions (ultrahigh dilutions) of antibodies to endothelial NO synthase (RAD of Abs to eNOS) (active pharmaceutical ingredient of drug “Impaza,” OOO “NPF “MATERIA MEDICA HOLDING”, Moscow, Russia) (2.5 mL/kg body weight) once a day, as a solution in distilled water (2.5 mL/kg body weight). The third group (PGK3 group) was given Subetta (5 mL/kg body weight) once a day, as a water solution (2.5 mL/kg body weight of RAD of Abs to *β*-InsR + 2.5 mL/kg body weight of RAD of Abs to eNOS). The dose of Subetta was the same as that used in the previous study in rats with streptozotocin-induced diabetes (5 mL/kg body weight), where drug showed significant antihyperglycemic efficacy [7]. So, Subetta contains RAD of Abs to *β*-InsR and RAD of Abs to eNOS. Ultrahigh dilutions of antibodies were obtained using routine methods described in the European Pharmacopoeia (6th Edition, 2007) as previously detailed [5] Antigen affinity purified rabbit polyclonal antibodies to *β*-subunit of insulin receptor or to endothelial nitric oxide synthase were produced from rabbit antiserum in accordance with the requirements imposed on animal immunosera for human use as described in the European Pharmacopeia (6th Edition, 2007). All dilutions were prepared in glass vials. Rabbit polyclonal antibodies to *β*-subunit insulin receptor or antibodies to endothelial NO synthase were mixed with a solvent (ethanol-water solution) and shaken for 1 min. to produce C1 dilution. All subsequent dilutions consisted of one part of the previous dilution to 99 parts of solvent (ethanol-water solution for intermediate dilutions and distilled water for preparation of the final dilution), with succession between each dilution. So, RAD of Abs to *β*-InsR, RAD of Abs to eNOS, and Subetta contain release-active dilutions of respective initial substances (or its combination in case of Subetta), which were diluted up to receiving mixture of final dilutions C12 + C30 + C200. Solutions were prepared in sterile conditions, avoiding direct intense light, and were stored at room temperature. RAD of Abs to *β*-InsR, RAD of Abs to eNOS, and Subetta were provided by OOO “NPF “MATERIA MEDICA HOLDING” as a ready-to-use solution in distilled water.

The fourth group (H_2_O) received vehicle only (distilled water; 5 mL/kg body weight) and was used as the control diabetic group for RAD of Abs to *β*-InsR, RAD of Abs to eNOS, and Subetta. The fifth group (Rosi) received Rosiglitazone (ref 223207-34-1, Interchim, France) (5 mg/kg body weight, in 5 mL/kg body weight) as a solution in 1% carboxy-methyl-cellulose (CMC) (ref 5678, Interchim, France). The sixth group (CMC) receiving carboxy-methyl-cellulose only (1% CMC, 5 mL/kg body weight) was used as the control diabetic group for Rosi. The drugs and the vehicles were orally administered (using a gastric tube) in one daily dose (at 9.00) for four weeks (d1 to d28). Animal body weight, food intake, water intake, and basal (at 9.00, i.e., in the nonfed state) plasma glucose and insulin levels were checked twice a week throughout the protocol. On d0 and d28, whole blood HbA1c was measured in each group. On d1 and d28, basal plasma glucagon, GLP-1, leptin, and adiponectin levels were measured in each group.

Oral glucose tolerance tests (OGTT, 2 g glucose/kg body weight) were sequentially performed on each rat from each group: two days before the first administration of any drug (d0), on the first day of treatment (d1) (at 12.00 i.e., 3 hours after the drug administration), and on the last day of the treatment period (d28) (at 12.00 i.e., 3 hours after the drug administration). Each OGTT was performed at 12.00 in nonanaesthetized rats fasted from 9.00 (postabsorptive state). Blood samples were collected sequentially from the tail vein before (0) and 5, 10, 15, 30, 60, and 120 min after glucose intake. They were then centrifuged and the plasma was separated. Plasma glucose concentration was immediately determined on a 10 *μ*L aliquot and the remainder was kept at −20°C until insulin radioimmunoassay. 

Plasma glucose was determined with a glucose analyser (Beckman). Immunoreactive insulin in the plasma was estimated with an ultrasensitive ELISA for rat (ref 80-INSRTU-E01 insulin kit from ALPCO/Eurobio). GLP-1 (ref YK160 GLP1 EIA kit from Yanaihara Institute/AbCys), glucagon (ref YK090 glucagon EIA kit from AbCys), leptin (ref 22-LEPMS-E01 leptin EIA kit from ALPCO/Eurobio), and adiponectin (ref 22-ADPRT-E01 adiponectin ELISA kit from ALPCO/Eurobio) were assayed from the same aliquot (50 microliters) of plasma. HbA1c (ref 280-008EX Micromat II from Bio-Rad) was measured from 10 microliters of whole blood. 

Insulin and glucose responses during the glucose tolerance test were calculated as incremental plasma insulin values integrated over the 120 min period following the glucose injection (AUC insulin; ng/mL/120 min) and corresponding incremental integrated blood glucose values (AUC glucose; g/L/120 min) (sum of value at *t*_*n*_− value *t*_0_, for *t*_*n*_ = 5, 10, 15, 30, 60, and 120 min).

Statistical analysis was performed with following software-R version: 2.13.1, RCOM server version: 2.1. All the results are presented as means ± S.E.M. and statistical significance of differences between means values was evaluated by Mann-Whitney and Wilcoxon tests for unpaired and paired data, respectively.

## 3. Results and Discussion

All rats entered the study survived until the end of the study. Weight gain and water intake in RAD of Abs to *β*-InsR, RAD of Abs to eNOS, Subetta, and CMC groups were similar to those of the control H_2_O group during the four-week period (data not shown). RAD of Abs to *β*-InsR, RAD of Abs to eNOS, and CMC had no significant effect on basal food intake (versus the H_2_O control group) while in Subetta group food intakes on d1 and d28 were slightly but significantly (*P* < 0.05) lower as compared to the H_2_O control group: 69 ± 1 g/kg/day versus 74 ± 1 g/kg/day and 66 ± 1 g/kg/day versus 71 ± 1 g/kg/day, respectively. In the Rosi group, weight gain of the GK rats was similar to that of CMC rats, whereas water and food intakes on d28 were significantly lower (*P* < 0.01) as compared to the CMC control group: 88 ± 2 mL/kg/day versus 62 ± 1 g/kg/day and 102 ± 3 mL/kg/day versus 71 ± 2 g/kg/day, respectively.

Chronic treatment with Subetta and RAD of Abs to *β*-InsR, but not with RAD of Abs to eNOS, prevented diabetes progression and significantly decreased plasma glucose as compared with baseline values (153 ± 4 dg/mL versus 168 ± 8 dg/mL (*P* < 0.01) and 147 ± 4 dg/mL versus 167 ± 3 dg/mL (*P* < 0.001), resp.) and with H_2_O control group as well in case of Subetta (147 ± 4 dg/mL versus 165 ± 4 dg/mL (*P* < 0.01)) (Table 1). Quite unexpectedly, CMC, used as a control for Rosi, exerted a slight but significant ameliorating effect on plasma glucose. This observation could reflect a delayed gastric emptying/intestinal absorption due to its high fibers content. Probably, it is the reason why Rosi exerts significant antihyperglycemic effect only on d1 as compare to CMC group. However, its effect still remained significant on d28 as compared to H_2_O group (*P* < 0.01).

Baseline and final values of HbA1c, insulin, GLP-1, adiponectin, leptin, and glucagon are shown in Table 2 and Table 3. CMC had no significant effect on the above-mentioned parameters as compared to the H_2_O control group. Animals in Rosi group as compared to CMC control group displayed considerably higher level of adiponectin (*P* < 0.001) on d1 and d28 (increased by 43% and 53%, resp.) and lower level of leptin on d1 (decreased by 45% on d28 (*P* < 0.01)). Treatment with RAD of Abs to *β*-InsR resulted in increasing of plasma GLP-1 by 39% on d1 and by 37% on d28 as compared to H_2_O control group (*P* < 0.05). RAD of Abs to eNOS significantly decreased plasma leptin by 17% on d28 only (*P* < 0.01 versus H_2_O control group).

**Figure 1:**
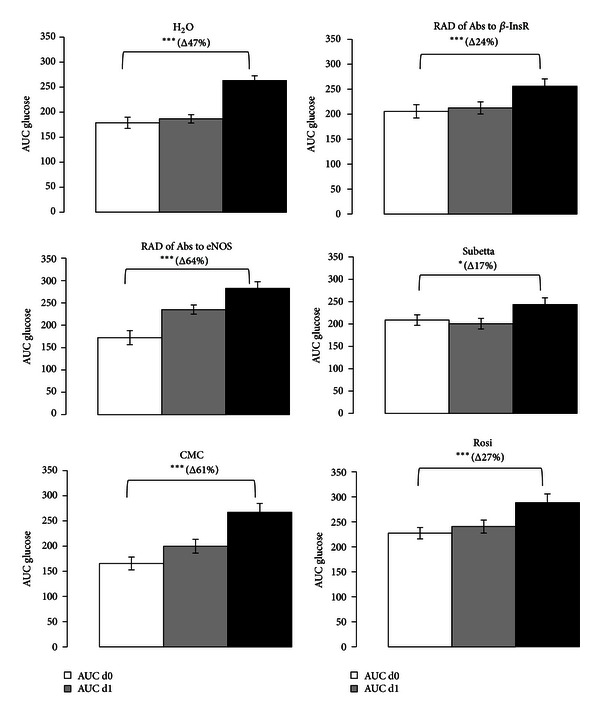
Glucose tolerance to glucose (2 g/kg per os) in adult diabetic male GK/Par rats before (d0), after acute treatment (d1), and after chronic treatment (d28) with RAD of Abs to *β*-InsR, RAD of Abs to eNOS, Subetta, H_2_O (distilled water), Rosiglitazone (Rosi), or carboxy-methyl-cellulose (CMC). On d1 and d28, drugs or vehicle were administrated at 9.00. OGTT were performed at 12.00 in nonanaesthetized rats fasted from 9.00 (postabsorptive state). Glucose responses during the glucose tolerance test were calculated as incremental plasma glucose values integrated over the 120 min period following the glucose injection (AUC glucose; g/L/120 min). Each point represents the mean ± S.E.M. of 12 observations/group. ***P < 0.001 versus the related d0-value within each group. **P < 0.01 versus the related d0-value within each group. *P < 0.05 versus the related d0-value within each group.

OGTT showed that glucose intolerance spontaneously deteriorated with aging (at least within the time-window 10–14 wks.) in the male GK/Par rats in both control groups (H_2_O and CMC) (Figure 1). Animals in RAD of Abs to *β*-InsR, Subetta, and Rosi groups exhibited significantly lower postoral glucose loading glucose levels than those in the controls: AUC glucose variations during the 28-days period were lower by 41% (*P* < 0.001), 59% (*P* < 0.05), and 41% (*P* < 0.05) as compared to respective controls (Figure 3). This establishes that both RAD of Abs to *β*-InsR and Subetta exert positive long-term effect upon glucose homeostasis in GK/Par rat model of T2D, which is comparable with Rosi effect. Herewith Subetta effect exceeds RAD of Abs to *β*-InsR effect.

**Figure 2:**
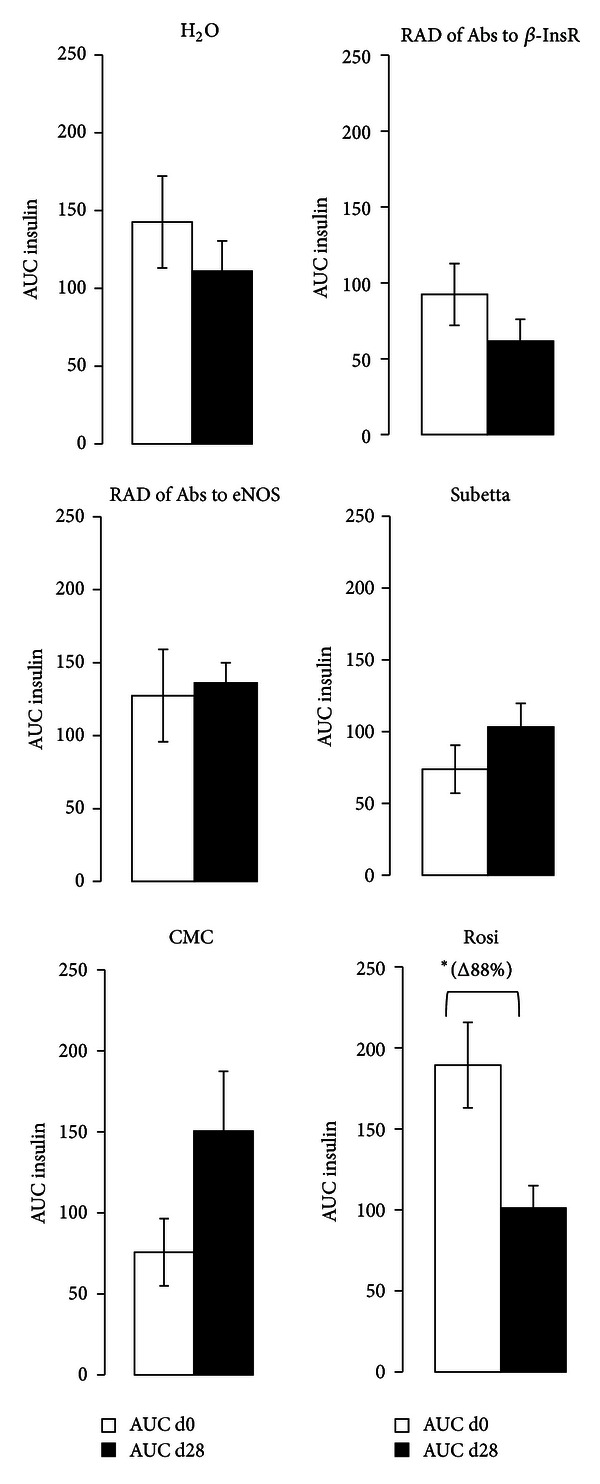
Insulin secretion in response to glucose (2 g/kg per os) in adult diabetic male GK/Par rats before (d0) and after chronic treatment (d28) with RAD of Abs to *β*-InsR, RAD of Abs to eNOS, Subetta, H_2_O (distilled water), Rosiglitazone (Rosi), or carboxy-methyl-cellulose (CMC). Insulin responses during the glucose tolerance test were calculated as incremental plasma insulin values integrated over the 120 min period following the glucose injection (AUC insulin; g/L/120 min). Each point represents the mean ± S.E.M. of 12 observations/group. *P < 0.05 versus d0-ROSI-treated GK/Par group.

**Figure 3:**
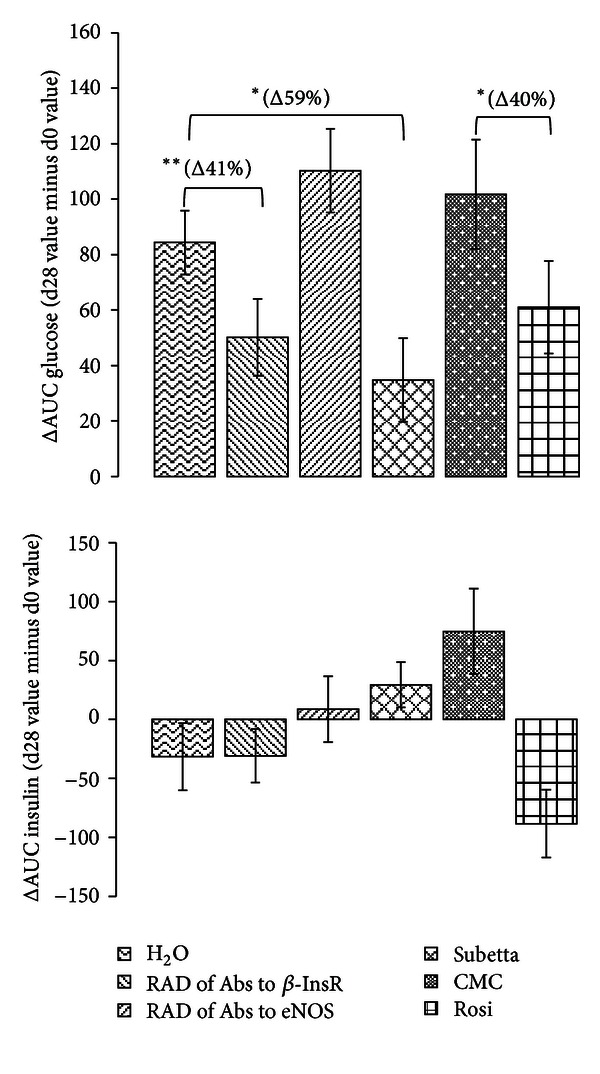
Time-related variations of AUC glucose and AUC insulin values in each group between d0 and d28. Such a calculation (d28 value minus d0 value) enables intergroup comparison. **P < 0.01 versus d0-H_2_O-treated GK/Par group. *P < 0.05 versus d0-CMC-treated GK/Par group.

The followup of glucose-induced insulin secretion (GSIS) showed that only treatment with Rosi resulted in lowering of insulin secretion in response to the oral glucose by the end of the four-week period as compared to baseline value (*P* < 0.05) (Figures 2 and 3).

Type 2 diabetes is a complex, heterogenous, and polygenic disease. The primary defects in insulin secretion and the development of insulin resistance contribute to the etiology of type 2 diabetes. Impaired postprandial insulin secretion because of functional defects and the loss of pancreatic *β*-cells leads to hyperglycemia and further decline in insulin sensitivity [20, 21]. Therefore, individuals with type 2 diabetes experience both reduced insulin secretion and insulin action. Thus, an animal model that mimics the pathogenesis and clinical features of human type 2 diabetes should have both of these traits. Among the animal models currently available, the Goto-Kakizaki rats exhibit inherited polygenic hyperglycemia, with low postprandial insulin secretion and insulin resistance, and they are widely used in experimental studies [22, 23].

In the present study, we evaluated the potential antidiabetic effects of two-component drug Subetta and its components RAD of Abs to *β*-InsR and RAD of Abs to eNOS in GK/Par diabetic rats. Herein, we showed that chronic oral administration of Subetta and RAD of Abs to *β*-InsR significantly attenuated fasting hyperglycemia and improved glucose homeostasis in GK/Par rats. The mechanism of Subetta action is not clear enough and seems to be complex because of the variety of the results received for its components and for the drug.

When we administered RAD of Abs to *β*-InsR for 4 weeks, it did not improve the HbA1c, insulin, adiponectin, leptin, and glucagon levels, but it did significantly increase GLP-1 levels at both stages of the treatment (on d1 as well as on d28). In spite of the significant increase of GLP-1 levels in RAD of Abs to *β*-InsR group, which is known to enhance satiety and causes inhibition of food intake and body weight gain in animal models and type 2 diabetes patients [24], RAD of Abs to *β*-InsR had no significant effect on basal food and water intake and weight of the GK rats. Subetta did not affect GLP-1; however, food intake was slightly but significantly lower in this group as compared to H_2_O control group. 

Treatment with RAD of Abs to *β*-InsR decreased plasma glucose starting from d20 to the end of the study. Nevertheless, these plasma glucose levels were still significantly higher than in H_2_O controls on d28 (*P* = 0.05). However, the glucose intolerance increased by 24% only in RAD of Abs to *β*-InsR group, a value significantly lower than that in the H_2_O control group. This effect could not be explained via increased GLP-1 level, which directly contributes to the improvement of glucose tolerance by inhibition of glucose production from liver and increasing of glycogen synthase activity [24]. GLP-1 remained unchanged in Subetta group, but the drug exerted a significant positive effect on the glucose intolerance in response to glucose load after chronic (28 days) administration too. More precisely, our data clearly demonstrate that Subetta effect exceeds RAD of Abs to *β*-InsR effect. It is important to notice that the improvement registered in Subetta and RAD of Abs to *β*-InsR groups was similar to that obtained in the Rosiglitazone-treated group under the same conditions. 

RAD of Abs to *β*-InsR and Subetta have shown only tendency to decrease leptin level at early (d1) stage of the treatment (as compared to H_2_O rats) (*P* = 0.06 and *P* = 0.05, resp.) meanwhile RAD of Abs to eNOS decreased it at both stages of the treatment, but significantly at later stage only. It is generally accepted that leptin has a potent inhibitory effect on insulin secretion from pancreatic *β*-cells *in vitro* and *in vivo* and has additional effect on reducing pre-proinsulin gene expression [25], but there were no effects on plasma insulin and insulin secretion levels in GSIS after neither RAD of Abs to eNOS, nor RAD of Abs to *β*-InsR, nor Subetta treatment. 

RAD of Abs to eNOS had not any significant effect on basal (nonfed state) plasma glucose level all along the treatment, despite of an isolated glucose elevation on d12, and glucose intolerance was as high as in H_2_O control group. Except for leptin, RAD of Abs to eNOS had no effect on any other basal plasma parameters related to diabetes status. Since RAD of Abs to eNOS is ineffective on GK diabetes, it can be inferred that the antidiabetic activity of Subetta does not rely on its “anti-eNOS” component, but could be related to Subetta “anti-*β*-InsR” component. On the other hand, an additive effect of RAD of Abs to eNOS might be caused by the improvement of oxidative stress and inflammation involved in diabetic pathology. RAD of Abs to eNOS is an active pharmaceutical ingredient of drug “Impaza” (OOO “NPF “MATERIA MEDICA HOLDING”, Moscow, Russia). It has been reported that it enhances the activity of endogenous endothelial NO synthase. Impaza revealed to be effective as monotherapy for erectile deficiency in the human and it also increases the efficacy of PDE5 inhibitors on combined treatment [4, 12, 13, 26]. The drug also showed endothelial protecting properties [1]. It seems that combination of RAD of Abs to *β*-InsR and RAD of Abs to eNOS resulting in combination of antidiabetic properties and endothelial protecting properties, respectively, creates the necessary and powerful prerequisites and reasonable background for Subetta application not only for diabetes management, but also for prevention of its complications.

Our findings suggest that Subetta and RAD of Abs to *β*-InsR action are mostly at the level of insulin action on the target tissues. Taking into account that Subetta (and RAD of Abs to *β*-InsR as it component) belong to the class of novel drugs and shares its common properties [3], such mechanism might be carried out by modulating effect of Subetta on the *β*-subunit of the insulin receptor regulating the insulin receptor's kinase activity and consequently activating receptor-associated signaling pathways [27]. Partly direct action of Subetta on insulin receptor has been recently confirmed *in vitro*, where it was shown that Subetta significantly stimulates adiponectin production by mature human adipocytes in the absence of insulin [5], which is known to enhance adiponectin regulation and secretion selectively in adipocytes [28]. In principle, ability of direct activation of insulin receptor and receptor-associated signal pathways in the absence of insulin was shown for L7 (Merck) [29]. Moreover in current *in vivo* study, Subetta showed tendency to increase plasma adiponectin (*P* = 0.07). 

Influence on adiponectin production could be an additional mechanism of Subetta action. The most significant role of adiponectin may be that of sensitizing the liver and muscles to the action of insulin in both humans and rodents. Adiponectin appears to increase insulin sensitivity by improving glucose and lipid metabolism [30]; adiponectin improves glucose metabolism apart from insulin signalling [31]; adiponectin regulates the expression of several pro- and anti-inflammatory cytokines. Its main anti-inflammatory function might be related to its capacity to suppress the synthesis of tumor necrosis factor alpha and interferon gamma and to induce the production of anti-inflammatory cytokines such as interleukin-10 and interleukin-1 receptor antagonist [32]; adiponectin has effects on *β*-cell function and survival, which are well known as key factors in the development of type 2 diabetes along with insulin resistance [25]. Finally, the results of the present study serve as a basis for further experiments which should be performed in order to test our hypothesis for the Subetta mechanism of action. 

Rosiglitazone, like other thiazolidinediones, reduces blood glucose levels by sensitizing insulin activity in target tissues, mainly by inhibiting lipolysis in adipose tissue and subsequent reduction of glucose production in the liver and enhancing insulin-mediated glucose disposal in skeletal muscle [33]. Our data in the Rosiglitazone-treated GK rats are consistent with this view, although we did not investigate directly glucose metabolism fluxes. They also match with previous reports in GK rats indicating that chronic troglitazone treatment improves their glucose tolerance through decreased hepatic glucose production and has a limited effect on peripheral insulin sensitivity [34]. Our data in the Rosiglitazone-treated GK rats also showed that plasma leptin and adiponectin levels are, respectively, decreased and increased. Leptin, the product of the ob gene, is a hormone secreted by adipocytes, and increased body fat content is closely correlated with the circulating plasma leptin levels [35, 36]. Leptin has been posited as a humoral signal from adipose tissue that acts on the central nervous system to reduce excess food intake and increase energy expenditure in a negative feedback manner [37, 38]. In the present study, acute exposure of Rosiglitazone significantly reduced plasma leptin levels. This reduction in leptin was associated with improvements in fasting hyperglycemia and glucose tolerance on the long term. In addition, we found that chronic Rosiglitazone tended to reduce food intake, which cannot be mediated by the decrease plasma leptin levels. Such pattern seems paradoxical since chronic Rosiglitazone is reported to increased plasma leptin levels due to the Rosiglitazone-induced increase in adiposity [39] through the activation of the adipocyte transcription factor peroxisome, proliferator-activated receptor-*γ* which stimulates adipocyte differentiation [40]. The paradoxical GK-response to Rosiglitazone could, at least partly, reflect a defective adipose tissue growth/differentiation in the GK model, as we previously suggested [41, 42]. In this study, chronic Rosiglitazone treatment significantly increased plasma adiponectin levels in the GK/Par rats. Finally, one may retain that both circulating leptin and adiponectin changes after Rosiglitazone in the GK/Par rats may contribute to the improvements of their hyperglycemia and insulin resistance.

## 4. Conclusion

Taken together, we conclude that Subetta and release-active dilutions of antibodies to *β*-subunit insulin receptor treatments are effective to significantly improve glucose homeostasis in GK/Par diabetic rats. Herewith Subetta effect exceeds, effect of, mentioned component. Moreover, since Subetta and release-active dilutions of antibodies to *β*-subunit insulin receptor behave as antihyperglycemic agents, it would be worthwhile to evaluate, after more prolonged administration, their effects on the residual pancreatic *β*-cell population, the low grade inflammation status of some tissues (pancreas, adipose tissue, liver), the circulating lipid status, and diabetic complications (kidney, heart, brain).

As such, Subetta and release-active dilutions of antibodies to *β*-subunit insulin receptor may be considered as new candidate antidiabetic drugs in the diabetic patients. Further studies are obviously needed to address more detailed information regarding the mechanisms of action for Subetta in treating diabetes.

## Figures and Tables

**Table 1:** Effect of test articles and appropriate controls on Goto Kakizaki/Par male rats basal (non-fed state) plasma glucose level (mg/dL; M ± SEM) during treatment period (28 days) (*n* = 12 in each group).

Groups	Plasma glucose level (mg/dL)							
	Day 1 (d1)	Day 4 (d4)	Day 8 (d8)	Day 12 (d12)	Day 16 (d16)	Day 20 (d20)	Day 24 (d24)	Day 28 (d28)
RAD of Abs to *β*-InsR	168 ± 8	167 ± 8	166 ± 8	165 ± 7	164 ± 6	153 ± 5**	156 ± 4*	153 ± 4**
RAD of Abs to eNOS	163 ± 10	159 ± 9	159 ± 8	179 ± 9*	169 ± 10	159 ± 10	167 ± 9	167 ± 11
Subetta	167 ± 3	165 ± 4	158 ± 5	163 ± 5	160 ± 7	152 ± 5*	158 ± 5	147 ± 4^∗∗∗##^
Rosi	169 ± 3^##^	154 ± 5	156 ± 5	153 ± 4	148 ± 3	150 ± 4	151 ± 5	145 ± 4
H2O	176 ± 6	176 ± 10	174 ± 12	169 ± 10	157 ± 6*	162 ± 10	165 ± 5*	165 ± 4*
CMC	186 ± 9	172 ± 8	167 ± 13	157 ± 8**	148 ± 5**	144 ± 4**	155 ± 4***	149 ± 5**

*P < 0.05 (versus d1)

**P < 0.01 (versus d1)

***P < 0.001 (versus d1)

^##^ *P* < 0.01 (versus control (H_2_O or CMC, resp.)).

**Table 2:** Whole blood HbA1c (%; M ± SEM), basal (non-fed state) plasma insulin (ng/mL; M ± SEM), and GLP-1 (ng/mL; M ± SEM) in Goto Kakizaki/Par male rats (*n* = 12 in each group).

Groups	Whole blood HbA1c level (%)		Plasma insulin level (ng/mL)		Plasma GLP-1 level (ng/mL)	
	Day 0 (d0)	Day 28 (d28)	Day 1 (d1)	Day 28 (d28)	Day 1 (d1)	Day 28 (d28)
RAD of Abs to *β*-InsR	8.38 ± 0.27^ND=4^	8.76 ± 0.26	2.7 ± 0.4	2.7 ± 0.4	7.70 ± 0.74^#^	7.24 ± 0.49^#^
RAD of Abs to eNOS	8.90 ± 0.21	9.85 ± 0.44*	2.8 ± 0.4	2.7 ± 0.4	6.10 ± 0.77	5.42 ± 0.66
Subetta	9.32 ± 0.50^ND=3^	8.87 ± 0.13	3.0 ± 0.3	2.2 ± 0.2**	5.70 ± 0.44	6.56 ± 0.61
Rosi	8.78 ± 0.24	9.18 ± 0.29	1.8 ± 0.4^##^	1.2 ± 0.1^∗#^	5.96 ± 0.52	5.51 ± 0.58
H2O	9.15 ± 0.17	8.77 ± 0.12	3.2 ± 0.4	2.4 ± 0.3***	5.30 ± 0.49	5.30 ± 0.50
CMC	9.30 ± 0.21^ND=6^	9.20 ± 0.39^ND=2^	4.0 ± 0.5	2.0 ± 0.3**	5.55 ± 0.47	4.58 ± 0.44

ND = 2, 3, 4 or 6: number of rats, for which whole blood HbA1c level (%) was not determined because of failure of the assays or insufficient number (volume) of samples

*P < 0.05 (versus d0 or d1, resp.)

**P < 0.01 (versus d1)

***P < 0.001 (versus d1)

^#^ *P* < 0.05 (versus control (H_2_O or CMC, resp.))

^##^ *P* < 0.01 (versus control (H_2_O or CMC, resp.)).

**Table 3:** Basal (non-fed sate) plasma adiponectin (ng/mL; M ± SEM), leptin (ng/mL; M ± SEM), and glucagon (ng/mL; M ± SEM) in Goto Kakizaki/Par male rats (*n* = 12 in each group).

Groups	Plasma adiponectin level (ng/mL)		Plasma leptin level (ng/mL)		Plasma glucagon level (ng/mL)	
	Day 1 (d1)	Day 28 (d28)	Day 1 (d1)	Day 28 (d28)	Day 1 (d1)	Day 28 (d28)
RAD of Abs to *β*-InsR	9539.2 ± 529.2	9751.4 ± 273.5	1669.2 ± 142.9	1664.1 ± 106.5	196.5 ± 25.0	230.3 ± 21.4
RAD of Abs to eNOS	10362.6 ± 433.9	10773.3 ± 612.5	1437.9 ± 86.5	1673.7 ± 106.2^∗∗##^	242.1 ± 29.0	184.2 ± 19.2
Subetta	10635.9 ± 416.5	11133.9 ± 567.4	1924.9 ± 328.4	1869.8 ± 236.4	233.0 ± 24.3	165.3 ± 25.5
Rosi	13816.2 ± 507.1^###^	14486.9 ± 436.4^###^	1293.0 ± 85.3^##^	1334.1 ± 61.6	152.0 ± 11.9	211.4 ± 28.7
H2O	10197.3 ± 392.9	9797.8 ± 482.0	2266.1 ± 210.1	2125.6 ± 254.5	199.9 ± 20.0	202.9 ± 21.0
CMC	9686.2 ± 415.5	9496.3 ± 356.0	2354.0 ± 413.9	2034.7 ± 307.0	205.4 ± 29.6	232.8 ± 49.3

**P < 0.01 (versus d1)

^##^ *P* < 0.01 (versus control (H_2_O or CMC, resp.))

^###^ *P* < 0.001 (versus control (H_2_O or CMC, resp.)).
