# Expression of Concern on “The Novel Oral Drug Subetta Exerts an Antidiabetic Effect in the Diabetic Goto-Kakizaki Rat: Comparison with Rosiglitazone”

**DOI:** 10.1155/2019/2938413

**Published:** 2019-06-16

**Authors:** Journal of Diabetes Research

*Journal of Diabetes Research* would like to express concern with the article titled “The Novel Oral Drug Subetta Exerts an Antidiabetic Effect in the Diabetic Goto-Kakizaki Rat: Comparison with Rosiglitazone” [1]. After issues were raised by several different parties [2–4] and we consulted editors, concerns were found with conflicts of interest, the nature of the therapy as related to homeopathy, text reuse from other authors, and the methods and reporting. Details are as follows.

## 1. Conflicts of Interest

Subetta (in Russian, Субетта), specifically “release-active dilutions of antibodies to *β*-subunit insulin receptor (RAD of Abs to *β*-InsR) and to endothelial nitric oxide synthase (RAD of Abs to eNOS)”, is a proprietary remedy owned by a company several of the authors are affiliated to, OOO “NPF” Materia Medica Holding; Sergey Tarasov is the Head of Research & Analytical Department, Oleg Epstein (also known as Epshtein) is the founder and CEO and holds related patents [5, 6], and Evgeniy Gorbunov is also affiliated. Though affiliations to and funding from OOO “NPF” Materia Medica Holding were stated in the article, this was not declared as a conflict of interest.

## 2. Homeopathy

The nature of Subetta as related to homeopathy should have been made clear. The serial dilutions used, C12, C30, and C200, make it likely none of the original substance was left in the preparations. At C12, one molecule might remain; below this, none are likely to remain.

Dr. Epstein and colleagues have published on this topic since the late 1990s, first referring to “ultra-dilute potentized antibodies” and later coining the term “release-active dilutions” (RAD). The authors say RAD is not homeopathy [7] and some particles may be retained even at ultra-dilutions [8], but the description of the preparation is clearly related to homeopathy and RAD have been previously reported as related to homeopathy by this group: for example, a 2006 article stated that “ultralow concentrations of the antibody were obtained using routine homeopathic methods” and referred to their previous work as “drugs obtained by homeopathic methods” [9], the patents notes that the treatment is “prepared according to homeopathic technology” [5] and “homeopathically potentized” [6], and a 2013 article by Dr. Epstein discusses homeopathy as a form of “release activity” and notes that production of RAD uses “Hahnemann's method” [10], i.e. homeopathic preparation, named after the founder of homeopathy Samuel Hahnemann. Despite concerns over the plausibility of ultra-dilute antibodies having activity, in 2016 the United Kingdom High Court overturned a decision of the hearing officer of the Intellectual Property Office to refuse patents to Dr. Epstein's products [11].

## 3. Text Reuse and Prior Work

The article reused around 250 words without citation from Tahara et al., 2011 [12]. The effects of rosiglitazone, a drug formerly used in diabetes treatment, in that previous diabetes mouse model were not the same as in the present study, in which it was used as a positive control. The authors have apologised for the lack of citation. An article from 2012 studying rosiglitazone in GK rats was also not discussed (Gao and Jusko, 2012) [13]. Those results of those two articles could have been compared to the current study, though the authors noted the different models, species, pathogenesis, doses, and duration of treatment, and that the aim of their study was not to study rosiglitazone.

This work is similar to a study published by one of the same authors, Dr. Epstein, in reference 7 (Kheyfets et al., 2012) [14], that used a different rat model of diabetes. Streptozotocin-induced diabetes was used in that study, while GK rats were used in this one. Though that article is cited there is no discussion of how the results compare; the authors argued during the review process that this was not necessary because the models are different.

## 4. Methods and Reporting

No blinding is reported, though the authors say the analysis was blinded.

There are no statistical adjustments for multiple hypothesis testing, though the authors provided an analysis adjusted using the Benjamini-Hochberg procedure that maintained statistical significance. Panchin et al. [2] argued, in the supplementary material, that the authors should have used a regression analysis and that after adjustment for the false discovery rate there is no significant effect of Subetta; the author Dr. Tarasov replied that the assumptions by Panchin et al. are not supported [8].

Samples of Subetta were stored at room temperature, but “storage at room temperature often leads to antibody degradation and/or inactivity, usually resulting from microbial growth” [15]. If the action of Subetta were dependent on antibody activity following ultra-dilution, storage at room temperature would have affected the results.

The abstract says fasting plasma glucose decreased in rosiglitazone versus vehicle, but this was not so when compared to the appropriate control, carboxy-methyl-cellulose (CMC). Therefore, the intended positive control, rosiglitazone, was no better than the negative control at reducing plasma glucose.

Some marginal, inconsistent, or negative results were not highlighted:

- The lack of effect of Subetta on adiponectin, leptin, or glucagon is not mentioned in the abstract
- No treatment affected HbA1c or GLP-1 other than marginal evidence of increased HbA1c with RAD Abs to eNOS and increased GLP-1 with RAD Abs to beta-insulin receptor versus water (Table 2);
- Only rosiglitazone increased adiponectin levels (Table 3); this is the opposite of an in vitro study by the authors, but this discrepancy is not discussed
- Rosiglitazone had lower leptin levels than vehicle, CMC (Table 3), but this is only indicated as significant at day 1 and not day 28, though the text indicates a significant reduction at day 28.
